# Specific functions of *Exostosin-like 3* (*EXTL3*) gene products

**DOI:** 10.1186/s11658-020-00231-y

**Published:** 2020-08-20

**Authors:** Shuhei Yamada

**Affiliations:** Department of Pathobiochemistry, Faculty of Pharmacy, Meijo University, 150 Yagotoyama, Tempaku-ku, Nagoya, 468-8503 Japan

**Keywords:** Exostosin-like 3 (EXTL3), Heparan sulfate (HS), Biosynthesis, Glycosaminoglycan, Regenerating islet-derived (REG) protein

## Abstract

*Exostosin-like 3* (*EXTL3*) encodes the glycosyltransferases responsible for the biosynthesis of the backbone structure of heparan sulfate (HS), a sulfated polysaccharide that is ubiquitously distributed on the animal cell surface and in the extracellular matrix. A lack of EXTL3 reduces HS levels and causes embryonic lethality, indicating its indispensable role in the biosynthesis of HS. EXTL3 has also been identified as a receptor molecule for regenerating islet-derived (REG) protein ligands, which have been shown to stimulate islet β-cell growth. REG proteins also play roles in keratinocyte proliferation and/or differentiation, tissue regeneration and immune defenses in the gut as well as neurite outgrowth in the central nervous system. Compared with the established function of EXTL3 as a glycosyltransferase in HS biosynthesis, the REG-receptor function of EXTL3 is not conclusive. Genetic diseases caused by biallelic mutations in the *EXTL3* gene were recently reported to result in a neuro-immuno-skeletal dysplasia syndrome. EXTL3 is a key molecule for the biosynthesis of HS and may be involved in the signal transduction of REG proteins.

## Introduction

Hereditary multiple exostosis (HME), also known as multiple osteochondromas, is a rare disorder occurring in approximately 1 in 50,000 individuals [1, 2]. It is characterized by cartilaginous or bony tumors, called osteochondromas or exostoses, which form within the perichondrium flanking the growth plates of the long bones, vertebrae, ribs and cranial base [3].

Previous studies on the genetic linkage for this disease reported some loci on the chromosomes, with mutation analyses identifying two genes as tumor suppressors: *exostosin 1* (*EXT1*) and *exostosin 2* (*EXT2*) [4–6]. Functional analyses showed that these genes encode the glycosyltransferases responsible for the biosynthesis of the backbone structure of heparan sulfate (HS), a sulfated polysaccharide that is ubiquitously distributed on the animal cell surface and in the extracellular matrix [7, 8]. Screening for proteins homologous to EXT1 and EXT2 identified the EXT-like proteins, EXTL1, EXTL2 and EXTL3, which are members of the EXT family (Table 1) [10–12]. These EXT-like proteins are also glycosyltransferases, and at least EXTL3 can catalyze the chain elongation reactions of HS polysaccharides [13, 14]. Although their involvement in HME has not yet been reported, some genetic disorders and traits related to these genes have been updated on OMIM (Online Mendelian Inheritance in Man). Table 1 shows the MIM (Mendelian Inheritance in Man) numbers of the EXT family.

**Table 1:** Human EXT family

Gene	Chromosomal location	Amino acid	Sequence identity with EXTL3 (%) [9]	mRNA accession number	MIM number
*EXT1*	8q24.11	746	26	NM_000127	133,700 215,300 608,177
*EXT2*	11p11.2	751	29	NM_000401	133,701 608,210 616,682
*EXTL1*	1p36.11	676	23	NM_004455	601,738
*EXTL2*	1p21.2	330	26	NM_001439	602,411
*EXTL3*	8p21.1	919	–	NM_001440	605,744 617,425

This review focuses on EXTL3, a critical enzyme for the biosynthesis of HS polysaccharides. A novel function for EXTL3 as a receptor for regenerating islet-derived (REG) proteins is also discussed and congenital diseases caused by mutations in the human *EXTL3* gene are described.

## Structure and functions of HS

**Fig. 1:**
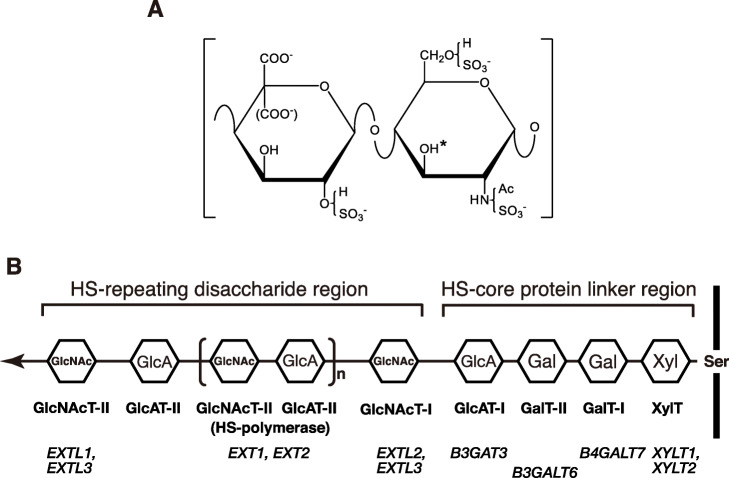
Structure and biosynthesis of HS. **a** – The structure of the repeating disaccharide unit in HS. The polysaccharide backbone of HS is composed of an alternating repeat of GlcA/IdoA and D-glucosamine (GlcN): -4GlcAβ/IdoAα1-4GlcNα1-. The amino group of GlcN is acetylated or sulfated. Hydroxy groups at the C6 of GlcN and the C2 of GlcA/IdoA may be sulfated. The hydroxy group of the C3 position (indicated by an asterisk) of *N*-sulfated/*N*-unsubstituted GlcN residue in specific saccharide sequences is possibly sulfated. **b** – A schematic drawing of the structure of HS polysaccharides. The enzyme responsible for the transfer of each monosaccharide residue is shown below the corresponding sugar residue. Gene(s) encoding each enzyme are also shown below. The arrow indicates the direction of biosynthesis. XylT, xylosyltransferase; GalT-I, galactosyltransferase I; GalT-II, galactosyltransferase II; GlcAT-I, glucuronyltransferase I; GlcAT-II, glucuronyltransferase II; GlcNAcT-I, GlcNAc transferase I; GlcNAcT-II, GlcNAc transferase II

HS is a linear polysaccharide composed of an alternating repeat of D-glucuronic acid (GlcA) and *N*-acetyl D-glucosamine (GlcNAc): -4GlcAβ1-4GlcNAcα1-. The amino group of the GlcNAc residue can be sulfated instead of acetylated. Some GlcA residues are C5-epimerized into iduronic acid (IdoA) residues. Hydroxy groups at the C6 position of GlcNAc/2-*N*-sulfated D-glucosamine and the C2 position of GlcA/IdoA may also be sulfated. A rare sulfation reaction occurs at the hydroxy group of the C3 position of 2-*N*-sulfated/2-*N*-unsubstituted glucosamine residue. These modifications provide structural diversity in HS chains (Fig. 1a), which contributes to their versatile functions, including the regulation of cell proliferation, cell differentiation and cell–cell recognition through interactions with bioactive proteins, such as growth factors, morphogens, cytokines, coagulation factors, proteases and extracellular matrix molecules [15, 16].

## Biosynthetic enzymes of HS

The biosynthesis of HS (Fig. 1b) is initiated by the xylosyltransferase-mediated transfer of a xylose (Xyl) residue from uridine diphosphate-xylose (UDP-Xyl) to a specific serine residue on a core protein [17]. Two galactose (Gal) residues are consecutively transferred from UDP-Gal to Xyl and Gal-Xyl on the core protein by galactosyltransferases I and II, respectively, to form a Gal-Gal-Xyl-core protein structure. The GlcA residue is transferred as the 4th sugar residue from UDP-GlcA by glucuronyltransferase I to form the so-called linkage tetrasaccharide region, GlcA-Gal-Gal-Xyl. The first GlcNAc residue is then transferred to the GlcA residue on this tetrasaccharide by the enzyme activity of GlcNAc transferase I from UDP-GlcNAc. GlcA transferase II and GlcNAc transferase II are respectively responsible for the transfer of GlcA and GlcNAc residues from UDP-sugar donors to form the repeating disaccharide region of HS, from the 6th sugar residue to the non-reducing end. This critical reaction to form HS polymers is catalyzed by the bifunctional enzymes, HS polymerases, which exhibit the activities of GlcA transferase II and GlcNAc transferase II [7]. HS polymerases are encoded by the *EXT1* and *EXT2* genes.

The EXT1 and EXT2 proteins form a hetero-oligomeric complex in vitro that leads to the accumulation of both proteins in the Golgi to exhibit stronger activities than monomer proteins [18, 19]. EXTL1, EXTL2 and EXTL3 also exhibit glycosyltransferase activities that are involved in the biosynthesis of HS. EXTL1 exhibits the enzymatic activity of GlcNAc transferase II, but is not an indispensable enzyme for the biosynthesis of HS [13]. Although other EXT family members are ubiquitously expressed in the human body and HS is systemically distributed, the expression of EXTL1 is restricted to the brain, liver and kidneys [20]. EXTL2 has been identified as the GlcNAc transferase I for HS biosynthesis in vitro [14]. However, it does not appear to be involved in the chain elongation of HS polysaccharides in vivo, but rather in the chain termination of HS because the knockdown of *EXTL2* increases the total amount of HS [21].

The enzymatic activity of EXTL3 was previously demonstrated to be that of GlcNAc transferases I and II using a recombinant protein [13]. Based on gene-silencing and overexpression experiments using mammalian cells, the in vivo activity of GlcNAc transferase I only appears to involve EXTL3. Overexpression of EXTL3 does not affect HS chain elongation [22]. By contrast, gene knockdown with EXTL3 siRNA resulted in an increased HS chain length [22], suggesting that when less acceptor substrate is available for HS polymerization, longer HS chains form. In addition, the activity of GlcNAc transferase II of EXTL3 was weaker than those of EXT1, EXT2 and the EXT1/EXT2 complex [22]. Recently, a large panel of Chinese hamster ovary cells with knockout or knock-in of the genes encoding most of the enzymes involved in glycosaminoglycan biosynthesis was engineered to generate a library of isogenic cell lines [23]. HS levels dramatically decreased in the Chinese hamster ovary cells with knockout of EXTL3. Therefore, EXTL3 seems to be responsible for initiating the chain elongation of HS polysaccharides. As with EXT1 and EXT2, the lack of EXTL3 leads to embryonic lethality, possibly because of failed HS biosynthesis [24].

It has also been reported that EXTL3 may control the modification of glucosamine residues in HS by regulating the activity of *N*-deacetylase/*N*-sulfotransferase-1 (NDST-1), which catalyzes the *N*-deactylation and *N*-sulfation of GlcNAc residues in HS [25, 26]. The formation of a complex between EXTL3 and NDST-1 reduces the *N*-sulfotransferase activity of NDST-1, but not its *N*-deacetylase activity, contributing to the generation of *N*-unsubstituted glucosamine residues in HS [27]. This structure shows an inhibitory effect on HS degradation against heparanase, which is a HS-specific hydrolase involved in HS catabolism. The exact function of EXTL3 in HS biosynthesis is still unclear.

## Characteristics of the EXTL3 protein

**Fig. 2:**
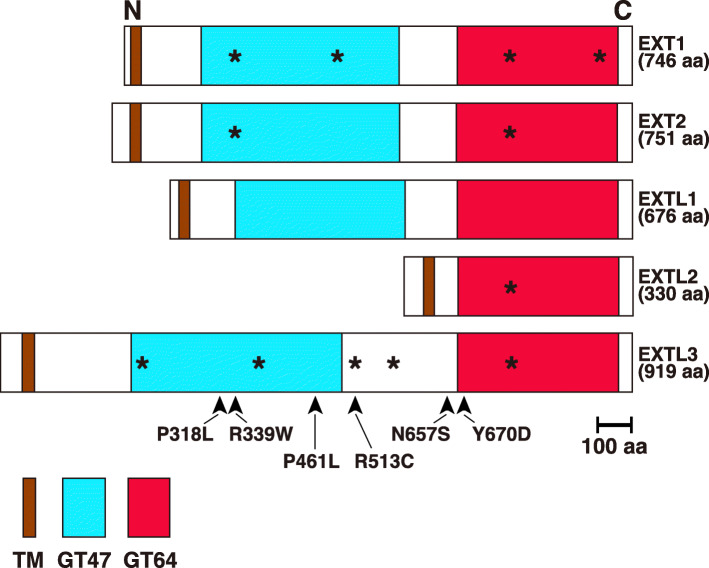
Schematic representation of human EXT family members. All are single-pass transmembrane proteins with a type II topology. They have short cytoplasmic, transmembrane (TM) and long Golgi luminal regions. Apart from EXTL2, two predicted glycosyltransferase domains belonging to glycosyl transferase family 47 (GT47) and GT64 are present. The former and latter are presumed to exhibit the enzymatic activities of GlcAT-II and GlcNAcT-II, respectively. EXTL2 is the shortest among the EXT family members, and only has the GT64 domain in the luminal region. Although EXTL3 exhibits GlcNAcT-I and GlcNAcT-II activities, but not GlcAT-II activity, it currently remains unclear whether the GT47 domain exhibits GlcNAcT activities. DXD (Asp-X-Asp) motifs in amino acid sequences are shown as asterisks, which are important for glycosyltransferases to interact with a Mn^2+^ cofactor that stabilizes binding of the diphosphate moiety of the UDP-sugars substrate. Arrowheads indicate the sites of mutations found in human genetic diseases (see Table 2)

The EXTL3 protein is composed of 919 amino acids, making it the longest member of the EXT family [9]. Percentage homologies between the amino acid sequence of human EXTL3 and those of human EXT1, EXT2, EXTL1 and EXTL2 were previously shown to be 26, 29, 23, and 26%, respectively (Table 1) [9]. EXTL3 is conserved from the common ancestor of all eumetazoans [9]. It is a type II transmembrane protein composed of a short cytoplasmic region, a transmembrane region, and a long Golgi luminal region, which has two glycosyltransferase domains (Fig. 2). The GlcA transferase II activity of EXTL3 has not yet been reported. It has been shown to exhibit GlcNAc transferase I and II activities. Since the glycosyltransferase domain on the N-terminal side (GT47) of EXT1/EXT2 was predicted to be GlcA transferase II, based on a bioinformatics analysis [32], the GT47 domain of EXTL3 may exhibit no enzyme activity. The GT64 domain in EXTL3 appears to have both GlcNAc transferase I and II activities. However, the possibility remains that the GT47 domain in EXTL3 may catalyze the GlcNAc transferase I reaction, i.e., the first transfer of a GlcNAc residue to the tetrasaccharide linkage region, initiating HS polysaccharide formation.

The conformation of the EXTL3 protein was recently characterized [32]. Awad et al. prepared the human EXTL3 protein without the sequence for the N-terminal cytosolic and transmembrane regions (EXTL3ΔN) and examined its stability and conformation. It was stable, with a high temperature needed to denature it. EXTL3ΔN has two *N*-glycans at Asn290 and Asn592. These are important for proper folding and secretion. The three-dimensional structure of EXTL3ΔN was examined using circular dichroism spectroscopy as well as solution small-angle X-ray scattering and dynamic light scattering. An extended structure consisting of two distinct regions was revealed, which was consistent with the bioinformatics data that suggest the presence of two glycosyltransferase domains.

## Analysis of Extl3-deficient mice

Conventional Extl3-deficient mice have been generated using the gene-targeting method. They were embryonically lethal at approximately 9 days post-coitum (dpc) (Table 2) [24]. HS was not synthesized in the embryos. Since difficulties have been associated with elucidating the tissue-specific functions of Extl3 due to the embryonic lethality of systemic Extl3-knockout mice, Extl3-floxed mice with two *loxP* sequences flanking exon 2 of the *Extl3* gene were generated. Some tissue-specific Extl3-deficient mice have been prepared using the Cre-lox recombination approach. Takahashi et al. created pancreatic β-cell-specific Extl3-knockout mice by mating Extl3-floxed mice with mice harboring the *Cre* transgene under the control of the rat insulin 2 promoter [24]. These mice had an abnormal islet morphology with reduced β-cell proliferation and glucose intolerance due to defective insulin secretion (Table 2), indicating the importance of HS or Extl3 in the regulation of postnatal islet maturation and normal insulin secretion.

**Table 2:** Loss of function mutations in *EXTL3* gene

Mutations	Phenotypes/Clinical Features	Reference
Systemic Extl3-deficient mouse	Embryonic lethality (9 dpc). No detectable HS by high performance liquid chromatography (HPLC) analysis.	[24]
Pancreatic β-cell-specific Extl3- deficient mice	Impaired postnatal islet maturation. Reduced HS level by immunostaining using an anti-HS antibody (3G10).	[24]
Podocyte-specific Extl3-deficient mice	Irregularities in the glomerular basement membrane and effacement of the foot processes. No increase in urinary albumin excretion. Reduced HS level by immunostaining using an anti-HS antibody, HS4C3, which recognizes the 3-*O*-sulfated domains of HS. HS4C3 (1:10), which especially binds to	[28]
Mutations in human EXTL3 c.1382C > T (p.Pro461Leu)	Lumbar gibbus, kyphoscoliosis, cervical malformations, hypoplastic odontoid peg with cervical instability, epiphyseal abnormalities, and intellectual disability. HS concentration decreased in the urine and serum, while was normal level in fibroblasts.	[29]
c.1537C > T (p.Arg513Cys)	Short stature, metaphyseal abnormalities of the long bones, early death, a lack of T cells, and liver cysts. HS concentration decreased in fibroblasts, while was normal level in the urine and serum.	[29]
c.1970A > G (p.Asn657Ser)	Cervical malformations, hypoplastic odontoid peg with cervical instability, metaphyseal abnormalities of the long bones, early death, idiopathic CD4^+^ lymphopenia, absolute lack of naive T cells, a typical skin rash, and liver cysts. HS concentration decreased in fibroblasts.	[29]
c.2008 T > G (p.Tyr670Asp)	Short stature, epiphyseal abnormalities, intellectual disability, a lack of T cells, and a typical skin rash. HS concentration decreased in fibroblasts as well as the urine and serum.	[29]
c.1015C > T (p.Arg339Trp)	Generalized platyspondyly with an increased intervertebral space, narrow sarco-ischiatic notches with a trident-shaped acetabula, and short and plump lim bones, metacarpals, and phalanges. Premature craniosynostosis. Narrowing of the cervical canal and severe narrowing of the laryngotracheal tract. Opisthotonus, hyperreflexia, and generalized seizures. Developmental delay, clonic arm movements, and nystagmus. Severe T-cell immunodeficiency. Loger HS chains with an aberrant sulfation pattern. This mutation does not affect the expression level of EXTL3 proteins in fibroblasts.	[30]
c.1382C > T (p.Pro461Leu)	Generalized platyspondyly with an increased intervertebral space, narrow sarco-ischiatic notches with a trident-shaped acetabula, and short and thick limb bones, metacarpals, and phalanges. Narrowing of the cervical canal. Muscular hypotonia and marked developmental delay. Severe T-cell immunodeficiency. This mutation does not affect the expression level of EXTL3 proteins in peripheral blood mononuclear cells.	[30]
c.953C > T (p.Pro318Leu)	Severe platyspondyly, kyphoscoliosis, pelvic distortion, constriction of the proximal femora, and brachydactyly. Glycosyltransferase activity of EXTL3 significantly decreased. This mutation does not affect protein stability of EXTL3.	[31]

Podocyte-specific Extl3-deficient mice have also been generated by crossing Extl3-floxed mice with mice expressing Cre recombinase under the control of the nephrin gene (*Npsh1*) promoter [28]. These mice showed significantly reduced HS levels in the glomerular basement membrane with an abnormal morphology (Table 2). However, urinary albumin excretion was not enhanced, indicating that the involvement of HS in the glomerular charge barrier was smaller than expected. Since other kidney cells synthesize HS, the functions of the glomerular filter barrier may be compensated by other HS-proteoglycans secreted by the surrounding cells. Further studies are needed to clarify the major negative charges of the kidney glomerulus.

We generated two Extl3-deficient mouse lines: glutamatergic neuron-specific using Cre recombinase under the control of the Ca^2+^/calmodulin-dependent protein kinase II gene promoter; and T-cell-specific using Cre recombinase under the control of the lymphocyte-specific protein tyrosine kinase gene promoter (unpublished results). Their phenotypes are currently being investigated.

## Novel function of EXTL3 as a cell surface receptor

As described above, EXTL3 functions as a GlcNAc transferase and is essential for the biosynthesis of HS. This has been established based in various studies using cellular and mouse models. Although the meanings have not been fully elucidated, EXTL3 seems to function as a cell surface receptor.

Kobayashi et al. identified Extl3 as a cell surface receptor of rat regenerating islet-derived (Reg) 1 protein, [33], which is an ortholog of human REG1A and REG1B. The Reg family comprises four groups of proteins: Reg1, Reg2, Reg3 and Reg4 [34]. REG1A and REG1B are expressed in humans, while Reg1 is expressed in rodents. There is no human ortholog of mouse Reg2. The Reg3 group includes the rodent Reg3a, Reg3b, Reg3g and Reg3d, and the human REG3A and REG3B. Rodent Reg4 or human REG4 have the lowest similarity with any other rodent or human Reg proteins.

Reg1 protein was initially discovered in regenerating islets of the pancreas. It has been shown to stimulate islet β-cell growth [35]. Some Reg family members are expressed in the gastrointestinal tract, brain, liver and skin, and they are implicated in cancer, inflammation or tissue injury. The signal of the Reg1 protein was reported to be mediated by cell surface EXTL3 [33].

When EXTL3 catalyzes the reaction for HS biosynthesis, it needs to be located at the Golgi apparatus. However, Kobayashi et al. showed that the cellular distribution of Extl3 was on the cell surface [33]. The overexpression of Extl3 in the rat insulinoma-derived β-cell line RINm5F increased its cell proliferation in response to a Reg1 protein, suggesting that Reg1 protein-dependent signaling is enhanced by the strongly expressed receptor Extl3 [33]. The expression of phosphorylated ERK and phosphorylated AKT was previously shown to be enhanced by stimulation with REG1A in HUVECs, indicating that the signaling pathway involving ERK and AKT was activated by REG1A via the EXTL3 receptor [36].

Most Reg1 studies have been performed using pancreatic cells, but it is also expressed in the central nervous system and its expression is developmentally regulated [37]. Acquatella-Tran Van Ba et al. examined the expression of the Extl3 protein in PC12 neuronal cells and β3-tubulin-positive rat hippocampal neurons using immunofluorescence, showing that it was mainly localized in the Golgi apparatus, but also co-localized with Reg1 protein at the plasma membranes of cell bodies and neurites [38]. Although the overexpression of Extl3 alone in PC12 cells did not modify the number of cells with longer neurites, adding Reg1 led to a 2-fold increase in the number of cells with longer neurites. This suggests that Reg1 effects on neurite outgrowth are mediated through its receptor Extl3. Furthermore, the proportion of cells with longer neurites was significantly smaller following the downregulation of Extl3 by shRNA than when the control non-effective shRNA was used.

The role of EXTL3 in Reg1-signaling has yet to be sufficiently demonstrated [34]. Direct interaction between Reg1 and AExtl3 has not been shown conclusively via immunoprecipitation or other methods. Although Mueller et al. performed a yeast two-hybrid analysis to find the binding partners of rat Reg1, the identified clones did not show homology with the *Extl3* gene [39]. Kadowaki et al. reported that EXTL3 does not seem to act upstream of kinases believed to be targeted by Reg1 [40]. Most importantly, a role of EXTL3 in intracellular signaling has never been confirmed. Although Mizuno et al. suggested that EXTL3 induces the NF-κB signaling through stimulation with TNF-α [41], a link between Reg1 and this signaling pathway has never been established. Therefore, evidence of a function for EXTL3 in Reg1 signaling is not only inconclusive but even conflicting.

The involvement of EXTL3 in the signaling of other Reg proteins has also been reported. Levetan et al. identified EXTL3 as a REG3A-binding protein on the cell surface and demonstrated that REG3A enhances EXTL3 translocation from the membrane to the nucleus [42]. Lai et al. demonstrated that REG3A is abundantly expressed in the epidermal keratinocytes of psoriasis patients and promotes wound re-epithelialization and psoriatic hyperproliferation [43]. Silencing EXTL3 inhibited the effects of REG3A on cell proliferation and differentiation. Furthermore, an antibody against EXTL3 abrogated the activity of REG3A. However, EXTL3 silencing did not decrease the effects of fibroblast growth factor 7, which requires HS as a co-receptor, indicating that REG3A acts through EXTL3 as its functional receptor expressed on the cell surface of keratinocytes independent of its glycosyltransferase activity.

Lai et al. also identified the downstream signaling molecule of EXTL3 as phosphatidylinositol 3-kinase (PI3K). REG3A was shown to activate the EXTL3-PI3K-AKT signaling pathway to regulate keratinocyte proliferation and/or differentiation [43]. However, the role of EXTL3 in REG3A signaling is also debatable [34]. The amount of HS on the keratinocyte cell surface and in the wound environment is very low, but the predominant glycosaminoglycan in the skin is dermatan sulfate, which can act as a coreceptor for fibroblast growth factor 7 [44]. Blocking EXTL3 did not affect REG3A-induced cell proliferation [45].

Epidermal growth factor receptor (EGFR) has been reported to be another candidate for the receptor for REG3A. Immunostaining showed that EGFR and REG3A can co-localize on SW1990 pancreatic adenocarcinoma cells and their complex could be detected using co-immunoprecipitation. The REG3A-enhanced expression of the cell cycle regulatory switch cyclin D1 could be negated by treatment with an EGFR inhibitor, suggesting the involvement of EGFR in REG3A signaling [45].

The involvement of EXTL3 in endometriosis, a benign chronic condition characterized by the existence of endometrial-like stroma and glandular tissue in extrauterine locations, was recently reported [46]. Unidentified factor(s) in the serum of patients interacted with EXTL3, resulting in increased colony formation in regenerating cell cultures. Although REG proteins were not examined in that study, EXTL3 may play a role in endometriosis as a membrane signaling molecule that interacts with these ligands. However, cell surface HS synthesized by EXTL3 may regulate the cell entry of viruses, potentially contributing to the pathogenesis of endometriosis.

## Congenital diseases caused by mutations in the human *EXTL3* gene

Genetic diseases caused by biallelic mutations in the *EXTL3* gene have been reported. Oud et al. found that homozygous missense mutations in *EXTL3*, including c.1382C > T (p.Pro461Leu), c.1537C > T (p.Arg513Cys), c.1970A > G (p.Asn657Ser) and c.2008 T > G (p.Tyr670Asp), resulted in a neuro-immuno-skeletal dysplasia syndrome [29]. Affected individuals presented with various skeletal abnormalities and neurodevelopmental defects. Some patients also showed severe combined immunodeficiency with a complete absence of T cells (Table 2). All four mutations were located in the luminal region, with the former two mutations and latter two near or inside the GT47 and GT64 domains, respectively (Fig. 2). The concentration of HS was lower in the fibroblasts of affected individuals, except for those from a patient with a homozygous missense mutation of c.1382C > T (p.Pro461Leu). The catalytic activities of GlcNAc transferases I and II in mutated EXTL3 proteins have not yet been assessed.

Volpi et al. also examined patients with severe skeletal dysplasia, T-cell immunodeficiencies and developmental delays (Table 2) [30]. The homozygous missense mutations in the *EXTL3* gene, c.1015C > T (p.Arg339Trp) and c.1382C > T (p.Pro461Leu), have been implicated in the pathogenesis of this disease. These two mutations are both located in the glycosyltransferase domain GT47. Abnormalities were detected in the production of HS by the fibroblasts of patients. However, the catalytic activities of GlcNAc transferases in mutated EXTL3 proteins have not yet been investigated.

We previously reported a novel type of spondylo-epi-metaphyseal dysplasia caused by biallelic *EXTL3* mutations [31]. Anomalies were observed in the spine, epiphyses and metaphyses of patients (Table 2). The mutation identified in the *EXTL3* gene was c.953C > T (p.Pro318Leu). It differed from those reported previously. The substituted residue in EXTL3 is highly conserved among diverse species [9]. We examined the GlcNAc tansferase II activity of the EXTL3 missense variant. The recombinant mutant protein exhibits markedly weaker activity than the wild-type enzyme, suggesting that the mutation reduces enzyme activity.

Based on the findings obtained from 14 patients from nine unrelated families, the phenotypic spectrum of the disease may now be defined [47]. Critical features are severe platyspondyly, brachydactyly and kyphoscoliosis. The majority of patients present with facial dysmorphisms, with upslanting palpebral fissures, frontal bossing, a prominent nose and a broad nasal tip. Truncal hypotonia and severe motor development delays are commonly observed. Some patients have a history of seizures and liver and kidney cysts. Several patients had T-cell lymphopenia, Omenn syndrome or eosinophilia, indicating that immune dysregulation is part of the phenotype of this disease. It currently remains unknown whether these phenotypes are due to abnormalities in the structure of HS and/or the receptor function of EXTL3.

## Concluding remarks

Several issues have yet to be resolved in studies on the EXTL3 protein. The role of EXTL3 in the biosynthesis of HS remains unclear. Although EXTL3 is considered to play a role in the initiation reaction of the biosynthesis of HS [22], this process does not appear to be inhibited at the transfer of the first GlcNAc residue in Extl3-deficient mice. This is because the phenotype of Ext1- or Ext2-deficient mice is more severe than that of Extl3-deficient mice. Both mice exhibit embryonic lethality, with Ext1- or Ext2-deficient mice and Extl3-deficient mice dying at 7.5 [48, 49] and 9 dpc [24], respectively. Weak and incomplete compensation by other glycosyltransferases such as Extl2 may prolong the survival of Extl3-deficient mice. However, these glycosyltransferases have not yet been identified. Therefore, the structures of presumably truncated HS chains synthesized by Extl3-deficient mice need to be elucidated.

The active domains for GlcNAc transferases in EXTL3 currently remain unknown. There are two predicted glycosyltransferase domains: GT47 and GT64 (Fig. 2). Based on previous studies on EXT1 [50], the GT64 domain is predicted to exhibit the activity of GlcNAc transferase II. However, it has not yet been established whether the GT47 domain in EXTL3 exhibits enzymatic activity, if this is GlcNAc transferase I activity, or whether the GT64 domain exhibits both GlcNAc transferase I and II activities. Therefore, further characterization of this enzyme is required. Mutations in *EXTL3* of the patient were found in the GT47 domain (Fig. 2), which is the predicted GlcA transferase II domain and may have no enzyme activity. However, HS concentrations were shown to be lower in some cases (Table 2).

Although EXTL3 functions as a receptor molecule, it has not yet been characterized in sufficient detail. More detailed structural information on the binding sites for ligands is needed, and the transduction of intracellular signaling warrants further study.

It is not clear whether symptoms of the diseases caused by mutations in the human *EXTL3* gene are due to the lack of HS or a deficiency in the cellular signaling of REG proteins through the EXTL3 receptor. This is because the GlcNAc transferase I activity of the mutated EXTL3 proteins has not been examined. In some cases, the level of HS in patient fibroblasts is not affected. Structural studies on HS in patients have been insufficient, but are essential for clarifying the underlying pathogenic mechanisms. A detailed characterization of Extl3-deficient mice may also resolve these issues.

## Data Availability

Not applicable.

## Abbreviations

EXT: Exostosin
EXTL: Exostosin-like
HS: Heparan sulfate
Gal: D-galactose
GlcA: D-glucuronic acid
GlcNAc: *N*-acetyl-D-glucosamine
IdoA: L-iduronic acid
Xyl: Xylose
REG: Regenerating islet-derived
UDP: Uridine diphosphate
